# Laccase-catalyzed decolorization and detoxification of Acid Blue 92: statistical optimization, microtoxicity, kinetics, and energetics

**DOI:** 10.1186/s40201-015-0183-1

**Published:** 2015-04-16

**Authors:** Shahla Rezaei, Hamed Tahmasbi, Mehdi Mogharabi, Alieh Ameri, Hamid Forootanfar, Mohammad Reza Khoshayand, Mohammad Ali Faramarzi

**Affiliations:** Department of Pharmaceutical Biotechnology, Faculty of Pharmacy and Biotechnology Research Center, Tehran University of Medical Sciences, P.O. Box 14155–6451, Tehran, 1417614411 Iran; Pharmaceutical Sciences Research Center, Tehran University of Medical Sciences, Tehran, 1417614411 Iran; Department of Medicinal Chemistry, Faculty of Pharmacy, Kerman University of Medical Sciences, Kerman, Iran; Department of Pharmaceutical Biotechnology, Faculty of Pharmacy, Kerman University of Medical Sciences, Kerman, Iran; Department of Drug and Food Control, Faculty of Pharmacy and Pharmaceuticals Quality Assurance Research Center, Tehran University of Medical Sciences, Tehran, 1417614411 Iran

**Keywords:** Enzyme Biocatalysis, Optimization, Waste Treatment, Bioremediation, Laccase, Decolorization

## Abstract

**Background:**

In recent years, enzymatic-assisted removal of hazardous dyes has been considered as an alternative and eco-friendly method compared to those of physicochemical techniques. The present study was designed in order to obtain the optimal condition for laccase-mediated (purified from the ascomycete *Paraconiothyrium variabile*) decolorization of Acid Blue 92; a monoazo dye, using response surface methodology (RSM). So, a D-optimal design with three variables, including pH, enzyme activity, and dye concentration, was applied to optimize the decolorization process. In addition, the kinetic and energetic parameters of the above mentioned enzymatic removal of Acid Blue 92 was investigated.

**Results:**

Decolorization of Acid Blue 92 was maximally (94.1% ± 2.61) occurred at pH 8.0, laccase activity of 2.5 U/mL, and dye concentration of 75 mg/mL. The obtained results of kinetic and energetic studies introduced the laccase-catalyzed decolorization of Acid Blue 92 as an endothermic reaction (Ea, 39 kJ/mol; ΔS, 131 J/mol K; and ΔH, 40 kJ/mol) with *K*_*m*_ and *V*_*max*_ values of 0.48 mM and 227 mM/min mg, respectively. Furthermore, the results of microtoxicity study revealed that the toxicity of laccase-treated dye was significantly reduced compared to the untreated dye.

**Conclusions:**

To sum up, the present investigation introduced the *Paraconiothyrium variabile* laccase as an efficient biocatalyst for decolorization of synthetic dye Acid Blue 92.

## Introduction

The wide usage of synthetic dyes in industries such as textile, paper, plastics, printing, leather, cosmetics, and pharmaceuticals has led to releasing dye containing effluents, rich in complex aromatic structures into the environment [1,2]. Almost all synthetic colorants especially the azo dyes − the most common dye group in textile dyeing processes − and/or their degradation products have been reported to be toxic, mutagenic, and carcinogenic [3]. Resistance of the environmentally hazardous dyes to light, biological treatment procedures, ozone, or other degradative environmental procedures is the main problem in the elimination of dyes discharged from wastewaters [4,5]. So, development of efficient and economical processes for treatment of the synthetic dyes still remains as a major challenge [1]. Among various physicochemical and biotechnological techniques, the enzymatic removal of synthetic dyes is the most preferred method due to its simplicity, efficiency at high and low pollutant concentration over a wide range of pH and temperature, low energy required, minimal impact on ecosystem, and less sludge production in the decolorization process [6-9].

Laccases (benzenediol:oxygen oxidoreductase, EC 1.10.3.2) are multi-copper containing oxidase mainly found in fungi, plants, and some bacterial strains [5,10]. The ability of laccases to oxidize a broad range of aromatic compounds such as benzenethiols, substituted phenols, and polyaromatic hydrocarbons (PAHs) in the presence of molecular oxygen as a co-substrate introduces this biotechnologically important enzyme as the first choice for xenobiotic removal experiments [11,12]. Decolorization and detoxification of synthetic dyes assisted by laccase and laccase-mediated system (LMS) have received great attention during two last decades [13,14]. However, high cost of enzymatic removal (due to low production yield) limits extensive application of laccases in xenobiotic elimination [15,16]. This constrain could be overcome via optimization of removal reaction conditions using statistical approaches [17,18].

The response surface methodology (RSM) which is utilized broadly in biotechnological processes, involves a collection of useful statistical and mathematical techniques for analyzing the causal relationship between independent variables, responses, and their interactions through the construction of polynomial mathematical models which leads to time and cost saving [19]. Several studies have been recently published on the potential applications of RSM in the enzymatic decolorization of reactive dyes such as Reactive Black 5, Reactive Red 239, Reactive Yellow 15, and Reactive Blue 114 [16,17].

In the present study, a D-optimal model for RSM as a very useful design method was applied to obtain maximal removal of Acid Blue 92 assisted by laccase. Furthermore, the kinetic and thermodynamic parameters of laccase-mediated dye removal reaction were investigated. The microtoxicity experiments were also performed in order to evaluate the toxicity of untreated and laccase-treated dye.

## Materials and methods

### Chemicals

**Figure 1:**
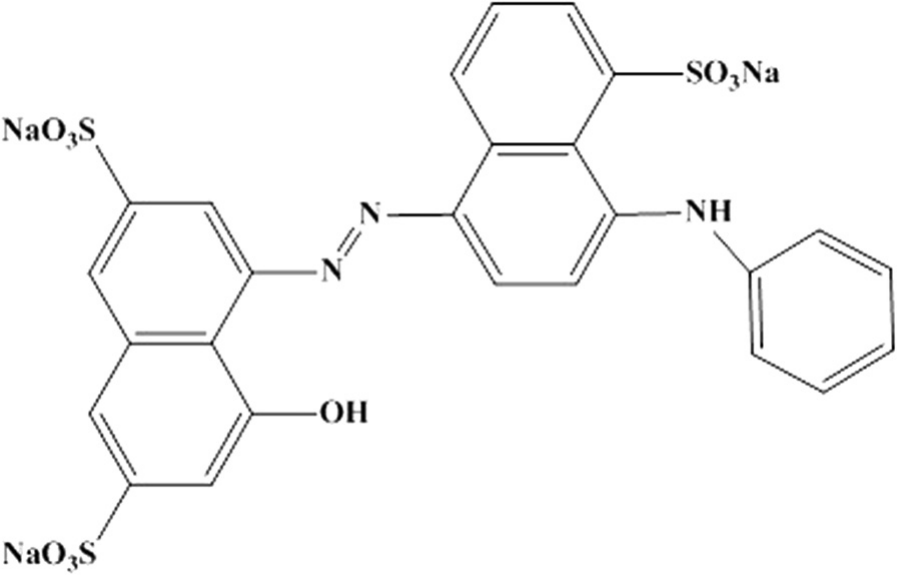
Chemical structure of Acid Blue 92.

2,2'-Azino-bis(3-ethylbenzthiazoline-6-sulphonate) (ABTS) was purchased from Sigma-Aldrich (St. Louis, MO, USA). Acid Blue 92 (Figure 1) was kindly donated by Alvan Sabet Co. (Tehran, Iran). All other chemicals and reagents were of the highest purity available. The extracellular laccase of *Paraconiothyrium variabile* (*Pv*L) was purified using the method previously described by Forootanfar et al. [20].

### Laccase assay

Laccase activity was determined using ABTS as the substrate [21]. The reaction mixture was prepared by adding 0.5 mL ABTS (5 mM) dissolved in 100 mM citrate buffer (pH 4.5) and 0.5 mL of enzyme solution followed by incubation at 40°C and 120 rpm. Oxidation of ABTS was monitored by increase in absorbance at 420 nm (ε_420_ = 36000 M^−1^ cm^−1^) using a UV/vis Spectrophotometer (UVD 2950, Labomed, Culver City, USA). One unit of laccase activity was defined as amount of the enzyme required to oxidize 1 μmol of ABTS per min [22].

### Dye decolorization experiments

After preparation of dye solution (concentration range of 100–200 mg/L) in citrate-phosphate buffer (0.1 M, pH range of 3.0 − 8.0), the purified *Pv*L (final activity of 1–2.5 U/L) was added to the reaction mixture and incubated at desired temperature (30–70°C) for 30–90 min followed by measuring the absorbance of the taken samples using a UV/visible spectrophotometer at maximum absorbance of applied dye (571 nm). Decolorization percentage was then calculated using the following equation: decolorization (%) = [*A*_*i*_ − *A*_*t*_/*A*_*i*_] × 100; where A_i_ is the initial absorbance of the reaction mixture and A_t_ is the absorbance after incubation time [1]. The negative control (reaction mixture containing the heat-inactivated enzyme) was prepared and incubated at the same conditions. All experiments were performed in triplicate and the means of decolorization percentages were reported.

### Experimental design and statistical analysis

#### Screening study

The screening experiment was designed based on the fractional factorial design method, which is a definite part of full factorial design matrix with two-level factor variations containing 2^k−p^ runs (1/2^p^ fraction of the 2^k^ design); where k and p are the number of independent variables and size of the fraction, respectively. Different factors including pH (A), temperature (B), enzyme activity (C), dye concentration (D), and incubation time (E) were implemented using 2^5–1^ fractional factorial design with resolution V. The design matrix was built using the statistical software package, Design-Expert (version 7.0.0; Stat-Ease, Inc., Minneapolis, Minnesota, USA). Factors and corresponding response presented in Table 1. All of the experiments were accomplished in triplicate and the averages considered as responses.

**Table 1:** **Level of independent variables in fractional factorial design**

**Variables**	**Symbol**	**Unit**	**Low level (−1)**	**High level (+1)**
pH	A	-	3	8
Temperature	B	°C	30	70
Enzyme	C	U/mL	1	2.5
Dye	D	mg/L	100	200
Incubation time	E	min	30	90

#### Optimization study

The D-optimal design developed to select the design points in a method that minimizes the variance associated with the estimates of coefficient in a specified model. The number of runs in a D-optimal design are less and do not rise as fast as the classical design with an increasing number of factors. The D-optimal design try to minimize the determinant of the (X’X)^−1^ matrix which lead to minimize the volume of the confidence for the coefficients and maximizes the determinant ‘information’ matrix (X’X); where “X” defined as a matrix containing the designed points generated by the computer to fulfill the D-optimally. The D-optimal designs, as an optimization method based on a chosen optimality criterion, used the determinant of the information matrix X’X. Maximizing the determinant of the information matrix (X’X) lead to minimizing the determinant of the matrix (X’X)^−1^ which beneficially keep the later calculations as short as possible. Finally, the Fisher’s test with P-values below 0.05 employed to evaluate the statistical significance of the effect of independent variables on the response using analysis of variance (ANOVA). While the multiple correlation coefficient (R^2^) and adjusted R^2^ used as quality indicators for the fit of second-order polynomial model equation, contour plots, and three-dimensional surface plots were used to graphically show the relationship and interactions between the coded variables and responses. The optimal condition for enzymatic decolorization process determined by solving the equation derived from the final quadratic model as well as grid search of the three-dimensional surface plots.

### Dye removal kinetics and energetics

#### Kinetics of decolorization

After performing of decolorization reaction in the presence of the dye concentrations (10–500 μM) at optimal pH and temperature, the velocity for different concentrations of dye was determined. Michaelis-Menten curve was then drawn by plotting the obtained initial velocity against dye concentrations. Calculation of *K*_*m*_ and *V*_*max*_ values were performed by fitting the data to the Lineweaver-Burk plot, resulting of the Michaelis-Menten plot conversion [1,2].

### Thermodynamics of decolorization

In order to evaluate the effect of temperature on removal of Acid Blue 92, decolorization experiment was performed at temperature range of 10–50°C and the obtained velocities (for each temperature) were plotted against initial dye concentrations. Thereafter, apparent first-order rate constant (K) for each temperature were calculated from the slope of each straight plot. The value of activation energy (E_a_) (kJ/mol) was then estimated from the slope of the linearized Arrhenius curve achieved by drawing the ln (K) versus 1/T (×10^3^ K^−1^): slope of Arrhenius plot = (−E_a_/R); where R is the gas constant (8.3145 J/mol K), and T is the absolute temperature (K). The enthalpy (ΔH) and entropy (ΔS) of decolorization reaction were estimated by using of Van’t Hoff plot which was drawn by plotting the ln (K_eq_) against 1/T (×10^3^ K^−1^). K_eq_ is the apparent equilibrium constant and was calculated from difference of initial dye concentration and residual dye concentration at equilibrium state, when the decolorization percentage become constant and remained dye have no longer changes with passing the time [23]. Finally, the Gibbs free energy (ΔG) was calculated using the equation of ΔG = ΔH – TΔS.

### Microtoxicity studies

In order to evaluate the toxicity of produced metabolite(s) following laccase treatment, a series of microtoxicity study was performed. Firstly, a preculture of three Gram-positive bacterial strains (*Micrococcus luteus* ATCC 10240, *Staphylococcus aureus* ATCC 6538, and *Bacillus subtilis* ATCC 6633) and three Gram-negative bacterial strains (*E. coli* ATCC 25922, *Pseudomonas aeruginosa* ATCC 9027, and *Salmonella typhi* ATCC 19430) was prepared by incubating of each bacterial strain in Muller-Hinton broth at 37°C and 150 rpm to reach the OD_600_ of 0.2. Consequently, the untreated dye solution (final concentration of 100 mg/L) and the sample obtained from enzymatic treatment of applied dye (performed at the optimized condition) was separately added to the prepared bacterial culture media and incubated at 37°C. Changes in the OD_600_ of each bacterial strain were then recorded every 2 h for 10 h. A negative control (cultivated bacterial strain in the absence of dye) was designed for each experiment. The percentage of growth inhibition (GI%) was defined as [(1 − D_600S_/OD_600C_) × 100]; where OD_600S_ is the OD_600_ of sample and OD_600C_ is the OD_600_ of control. All experiments were performed in triplicate and mean of the obtained results was reported.

## Results and discussion

### Fractional factorial design for screening of important variables

**Figure 2:**
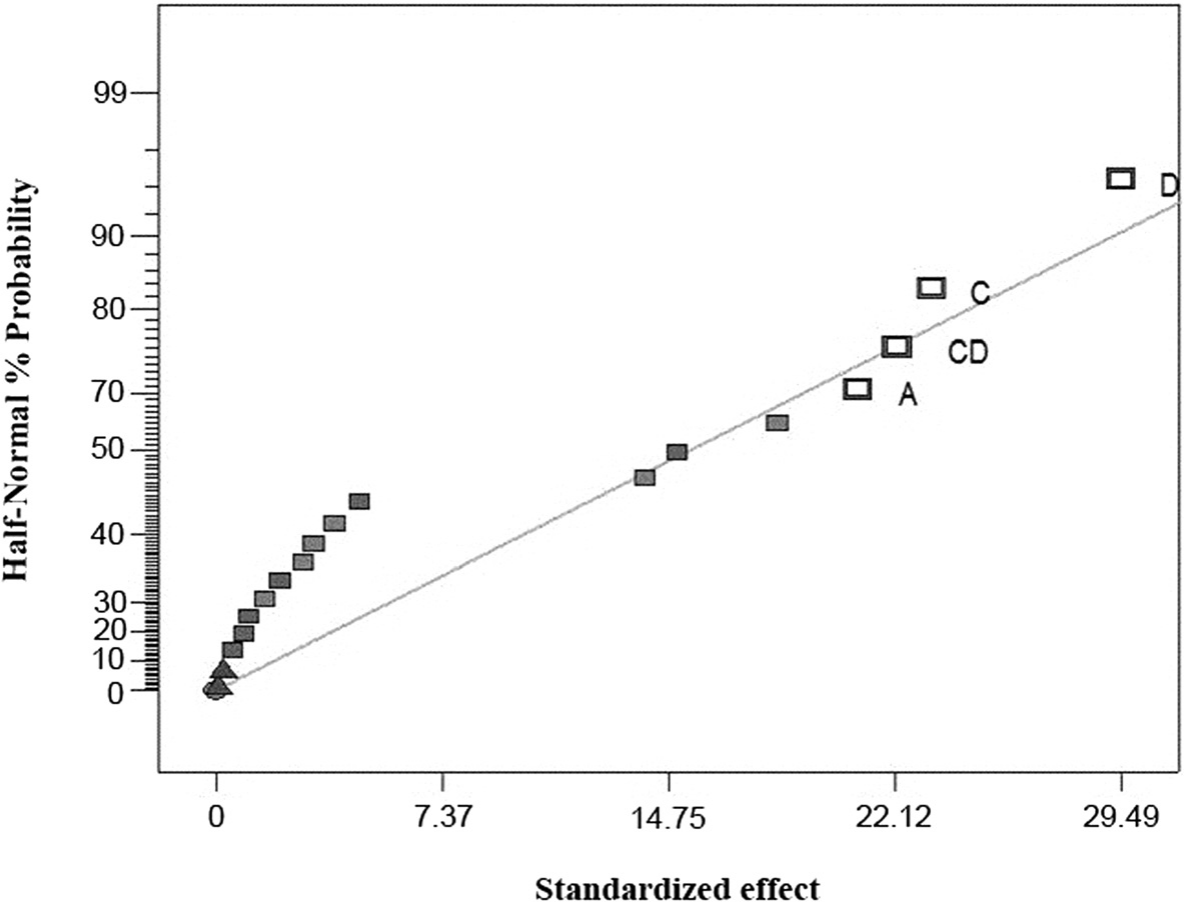
Half-normal probability plot for statistical analysis of fractional factorial design. A: pH; C: Enzyme; D: Dye.

In a preliminary study, fractional factorial design was used to evaluate the influence of five factors including pH (A), temperature (B), the enzyme activity (C), dye concentration (D), and incubation time (E) on the decolorization yield in order to select the most effective variables on laccase-catalyzed decolorization of Acid Blue 92. The respective high (+1) and low (−1) levels for each coded factor defined in Table 1. A total of 20 runs and the related responses with combination of five mentioned variables were presented in Table 2. The half-normal probability plot employed as a graphical tool for estimation the effect of each factor alone and in combination with other factors on decolorization percentage is illustrated in Figure 2. The large distance of pH (A), enzyme activity (C), and dye concentration (D) from zero in half-normal plot, indicated that these factors significantly affected the decolorization process (Figure 2). In addition, considerable interaction between enzyme activity (C) and dye concentration (D) emerged from half-normal probability plot (Figure 2). In the study of Khouni et al. [24], who applied central composite design (CCD) matrix and RSM for optimization of laccase-mediated decolorization of Black Novacron R and Blue Bezaktiv S-GLD 150, two variables including pH and temperature positively affected decolorization process while the third parameter (laccase activity) did not influence dye removal. Daassi et al. [25] reported the significant effect of four factors (laccase activity,1-hydroxybenzotriazole concentration, dye concentration, and reaction time) on decolorization of Acid Orange 51 assisted by crude laccase from *Trametes trogii* using a three-level Box-Behnken factorial design combined with response surface methodology to reach 87.87 ± 1.27 decolorization percent.

**Table 2:** **Fractional factorial design matrix and their observed responses for laccase-assisted decolorization of Acid Blue 92**

**Run no.**	**pH**	**Temperature (°C)**	**Enzyme activity (U/mL)**	**Dye concentration (mg/L)**	**Incubation time (min)**	**Response (decolorization%)**
1	5.5	50	1.00	100	60	13.62
2	5.5	50	1.00	100	60	16.60
3	8.0	30	2.50	50	90	16.28
4	8.0	70	2.50	200	90	4.64
5	8.0	30	0.25	200	90	6.94
6	3.0	70	2.50	200	30	10.55
7	3.0	30	2.50	200	90	6.65
8	3.0	30	2.50	50	30	92.66
9	8.0	30	2.50	200	30	5.27
10	3.0	70	0.25	50	30	22.90
11	8.0	30	0.25	50	30	13.31
12	3.0	70	2.50	50	90	92.45
13	3.0	30	0.25	50	90	14.57
14	3.0	70	0.25	200	90	5.89
15	3.0	30	0.25	200	30	7.87
16	8.0	70	0.25	50	90	3.81
17	8.0	70	2.50	50	30	32.40
18	8.0	70	0.25	200	30	3.65
19	5.5	50	1.00	100	60	22.25

### D-optimal design response surface methodology

**Figure 3:**
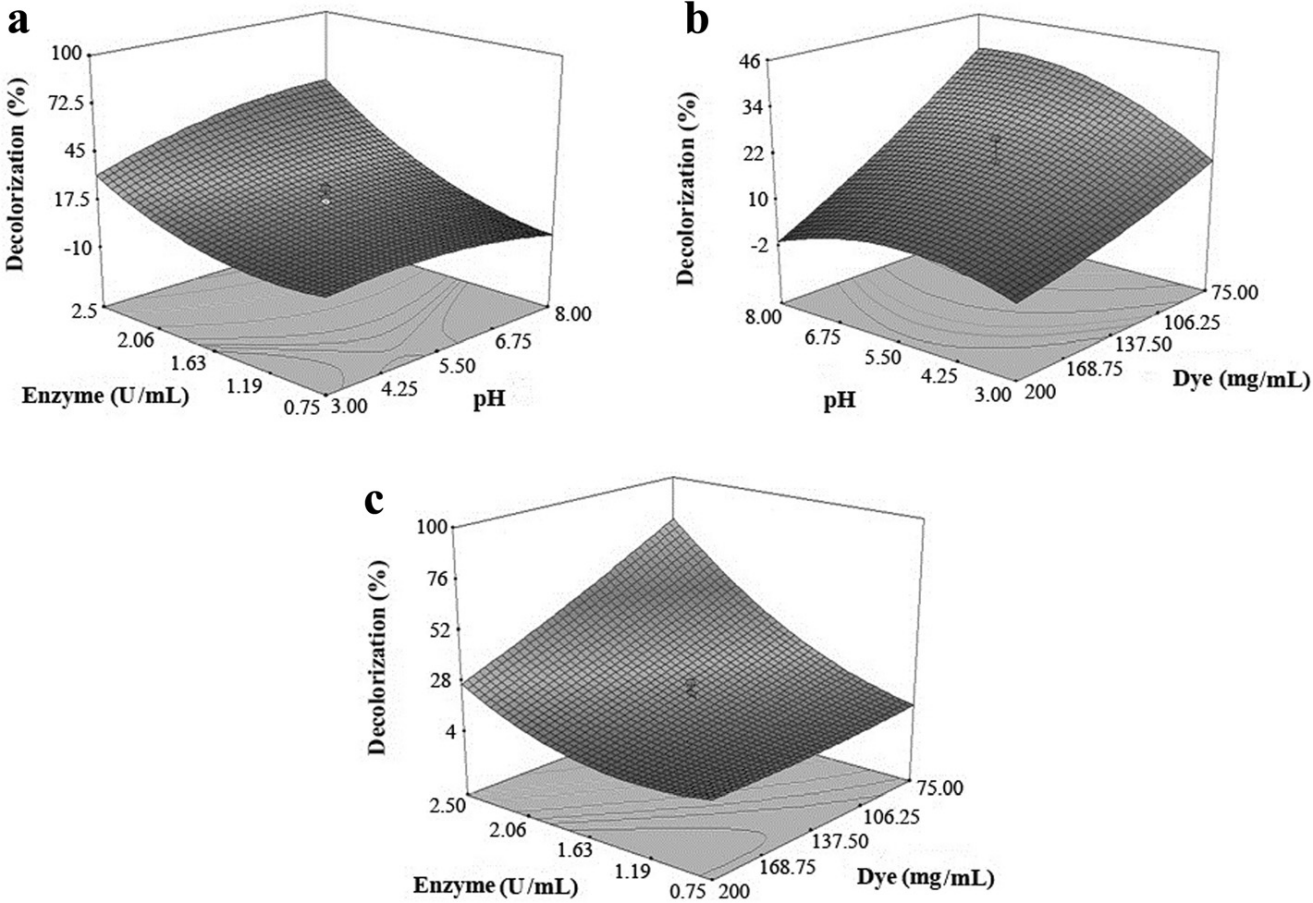
Response surface plot indicating the effects of interactions between **a)** pH and the enzyme activity, **b)** pH and the dye concentration, **c)** the enzyme activity and the dye concentration.

Based on the results of the fractional factorial design, three variables, including pH (A), enzyme activity (C), and dye concentration (D) were found to have a greater influence on the decolorization of Acid Blue 92 assisted by laccase. Therefore, the D-optimal RSM exploited to optimize the affecting decolorization factors. The responses generated from performing a total of 20 runs are presented at Table 3. Table 4 demonstrates the quadratic model as the most suitable model for the enzymatic decolorization of Acid Blue 92. The analysis of variance for the quadratic model is shown in Table 5. The model F-value (16.08) shows the significance of the model. In addition, Table 5 indicated pH (A), dye concentration (D), and their interactions; AC, CD, A^2^ and, C^2^ as significant model terms (P-value < 0.05). The quadratic model explained the statistical relationship between the selected variables and the response in terms of coded factors was best fitted with the following equation.

1 $$ Y = 18.27 + 3.99A + 21.55C\hbox{--} 13.5D + 8.59\  AC\hbox{--} 5.16\  AD\hbox{--} 12.37CD\hbox{--} 6.17{A}^2 + 11.42{C}^2 + 1.56{D}^2 $$

where Y is the response (yield of decolorization) and A, C, and D are pH, enzyme activity, and dye concentration, respectively. In order to investigate the relation between the independent variables and the responses, contour plots generated by RSM using the Design-Expert software. The response surface plots (Figure 3) present the decolorization of Acid Blue 92 as function of two variables, while the third one kept at a constant level. Surface plots demonstrate an increase in dye decolorization with raising the pH value. Figure 3a and Figure 3c clearly show that the decolorization percentage influenced by small alterations of the enzyme activity.

**Table 3:** **D-optimal design matrix containing various conditions and related responses**

**Run no.**	**pH**	**Enzyme activity (U/mL)**	**Dye concentration (mg/L)**	**Response (decolorization%)**
1	8.00	0.75	75.00	4.4
2	1.30	1.63	137.50	2.1
3	5.50	3.10	137.50	99.3
4	3.00	0.75	200.00	5.3
5	5.50	1.63	137.50	19.2
6	5.50	1.63	137.50	14.4
7	3.00	0.75	75.00	11.8
8	5.50	1.63	137.50	15.5
9	3.00	2.50	200.00	10.3
10	3.00	2.50	75.00	42.2
11	5.50	1.63	32.39	58.1
12	5.50	1.63	137.50	16.3
13	5.50	1.63	137.50	17.3
14	8.00	2.50	200.00	14.4
15	9.70	3.25	137.50	6.1
16	8.00	2.50	75.00	92.1
17	5.50	1.63	242.61	4.3
18	5.50	1.63	137.50	18.7
19	8.00	0.75	200.00	0.8
20	5.50	0.15	137.50	4.4

**Table 4:** **Sequential model sum of squares for D-optimal design**

**Source**	**Sum of squares**	**D**_**f**_	**Mean squares**	**F-value**	**P-value**
Mean vs Total	10503.78	1	10503.78	-	-
Linear vs Mean	9046.07	3	3015.36	8.54	0.0013
2FI vs Linear	2028.02	3	676.01	2.43	0.1121
Quadratic vs 2FI	2671.26	3	890.42	9.38	0.003
Cubic vs Quadratic	820.89	4	205.22	9.57	0.0089
Residual	128.66	6	21.44	-	-
Total	25198.68	20	1259.93	-	-

**Table 5:** **Analysis of variance for D-optimal design**

**Source**	**Sum of squares**	**D**_**f**_	**Mean squares**	**F-value**	**P-value**
Model	13745.35	9	1527.26	16.08	<0.0001
A-A	217.65	1	217.65	2.29	0.1610
C-C	6340.77	1	6340.77	66.78	<0.0001
D-D	2487.64	1	2487.64	26.20	0.0005
AC	590.82	1	590.82	6.22	0.0317
AD	213.31	1	213.31	2.25	0.1648
CD	1223.89	1	1223.89	12.89	0.0049
A^2^	548.32	1	548.32	5.77	0.0371
C^2^	1878.05	1	1878.05	19.78	0.0012
D^2^	34.93	1	34.93	0.37	0.5577
Residual	949.55	10	94.95	-	-
Lack of fit	883.05	5	176.61	13.28	0.0065
Pure error	66.5	5	13.3	-	-
Cor total	14694.9	19	-	-	-

### Optimization

By solving the equation (1) and analyzing three dimensional response surface graphs (Figure 3), the optimal levels for pH, enzyme activity, and dye concentration were found to be 8.0, 2.5 U/L, and 75 mg/L, respectively. The yield of laccase-catalyzed decolorization of Acid Blue 92 in optimal condition was 94.1% ± 2.61. The obtained results introduced pH as the most relevant factor for the enzymatic decolorization of Acid Blue 92. Low yield of decolorization achieved at acidic pH, while pH 8.0 was the optimal pH where maximum decolorization occurred. In general, most of fungal laccases optimally work at acidic pH levels [26]. For example, the crude laccase of *Ganoderma lucidum* preferred the acidic range for dye elimination and its decolorization activity decreased significantly at pH levels above 6.0 [16]. However, Tavares et al. [17] reported that more than 80% decolorization of Reactive Blue 114 occurred at pH 7.0–7.5. The maximum removal of Azure B using the purified laccase of *Trametes trogii* BAFC 463 mediated by 1-hydroxybenzotriazole was achieved at pH 7.0 [18]. In contrast, the laccases originated from bacterial strains maximally work at alkaline pH values. For instance, maximum decolorization activity of the purified laccase of *Bacillus pumilus* strain W3 was achieved at pH 9.0 [27]. The effect of the enzyme activity and dye concentration on decolorization of Acid Blue 92 at pH 8.0 (Figure 2c) revealed that decolorization decreased with increasing dye concentration, but increased with raising the activity of laccase. The results of the present study showed that more than 75% of the decolorization obtained at dye concentration of 75 mg/L and laccase activity of 2.5 U/L. Roriz et al. [15] reported that increasing of laccase activity (the crude laccase from *Trametes pubescens*) positively affected the decolorization of Reactive Black 5. Ashrafi et al. [1] demonstrated significant increase in removal of thirteen synthetic dyes when laccase activity increased in the range of 0.025–0.1 U/L.

### Validation of model

In order to determine the correctness of the model, five verification experiments were performed using the statistically optimal condition. The results showed maximum decolorization efficiency of 90.2%, which is about 99% of the predicted value, implying a strong similarity between experimental and predicted values calculated from the model that confirms the precision and validity of the model.

### Kinetics and energetics of decolorization

#### Kinetic study

**Figure 4:**
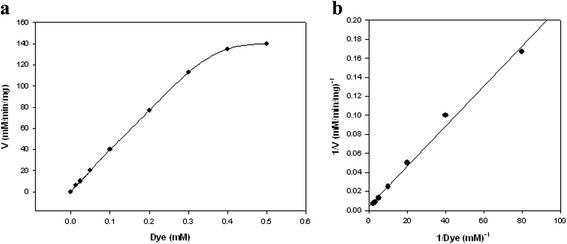
Kinetic study. **a)** Michaelis-Menten plot, **b)** Lineweaver-Burk plot.

The linear relationship between the initial velocity and dye concentration (Figure 4) indicated that the decolorization is a first order reaction. Based on the Michaelis-Menten and Lineweaver-Burk plots for enzymatic decolorization of Acid Blue 92 (Figure 4a and Figure 4b), the *K*_*m*_ and *V*_*max*_ values were 0.48 mM and 227 mM/min mg, respectively. In the study of Ashrafi et al. [1] who investigated on the ability of laccase for decolorization of a number of synthetic dyes, the lowest *K*_*m*_ (14.8 μM) found for the anthraquinone-based dye of Disperse Blue 56 followed by the monoazo dye of Acid Red 18 and the triazo dye of Direct Blue 71 with *K*_*m*_ value of 25 μM and 800 μM, respectively. In general, the azo dyes that are classified as monoazo, diazo, and triazo, have been identified as recalcitrant colorants for oxidation using laccases compared to that of anthraquinone-based dyes [1,3].

#### Energetic study

**Figure 5:**
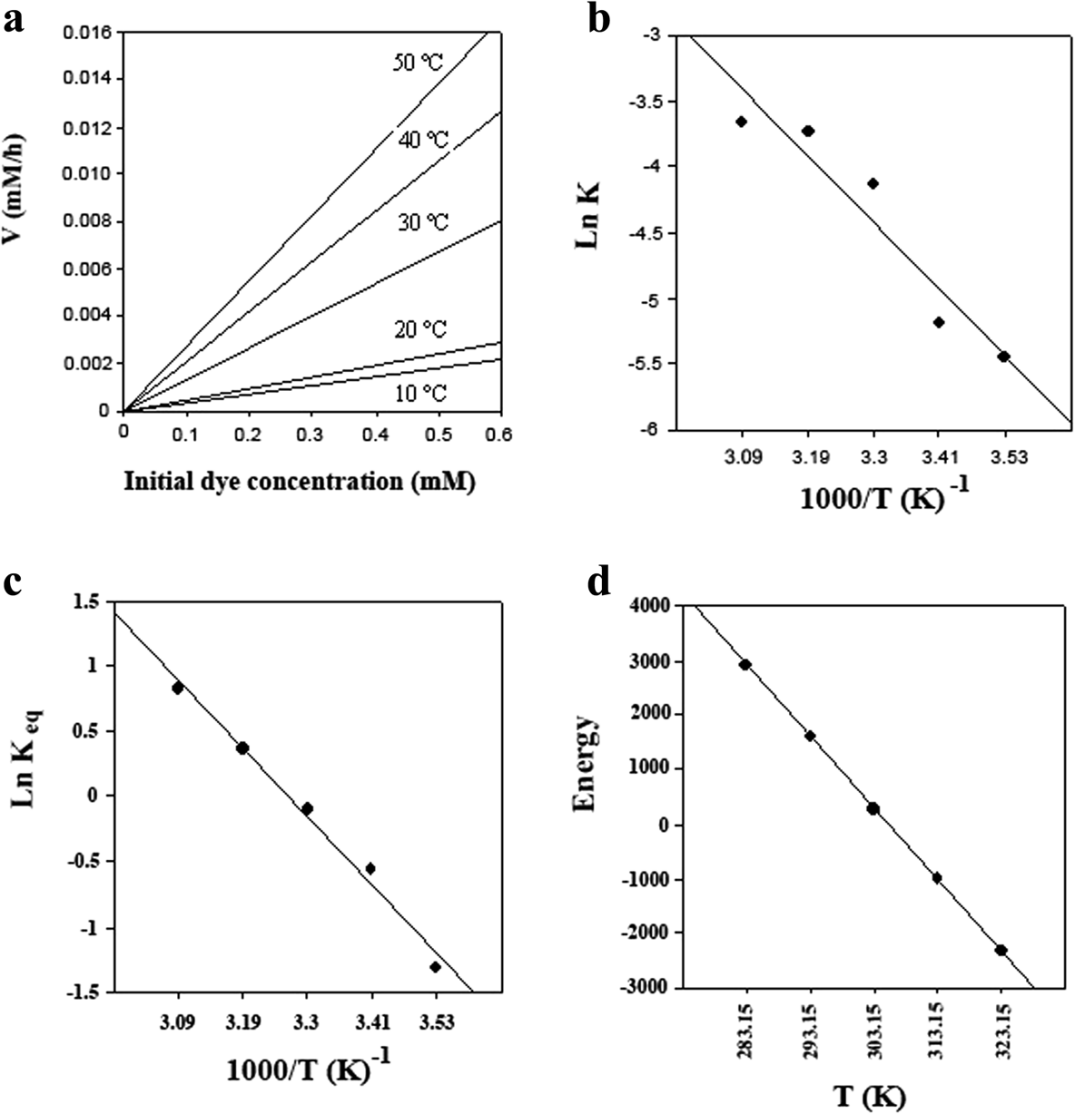
Energetic Study. **a)** dependence of decolorization rate on temperature (10–50°C), **b)** Arrhenius plot, **c)** van’t Hoff plot and, **d)** Gibbs free energy changes plot.

Energetic results are illustrated in Figure 5. A significant effect of temperature on decolorization of Acid Blue 92 was observed, as decolorization rate increased from 10 to 50°C. The activation energy (obtained from the slope of Arrhenius plot, Figure 5a) was 39 kJ/mol, which is typical for the decolorization reaction. The calculated values for ΔH and ΔS were 40 kJ/mol and 131 J/mol K, respectively. The positive sign of ΔH (Figure 5b) or the negative slope of van’t Hoff plot (Figure 5b) implies that the reaction is endothermic.

#### Dye toxicity

The obtained results of toxicity evaluation of untreated and laccase-treated dye solution showed that when Acid Blue 92 was used, the calculated GI% in the presence of *M. luteus*, *S. aureus*, *B. subtilis*, *E. coli*, *P. aeruginosa*, and *S. typhi* was found to be 60 ± 1.9, 49 ± 1.4, 51 ± 1.3, 42 ± 1.1, 30 ± 1.1, and 37 ± 0.8, respectively. However, the GI% for laccase treated dye solution was 31 ± 3.9, 34 ± 2.8, 27 ± 2.5, 20 ± 2.3, 17 ± 0.4, and 14 ± 0.9 in in the presence of *M. luteus*, *S. aureus*, *B. subtilis*, *E. coli*, *P. aeruginosa*, and *S. typhi*, respectively, which was significantly lower for all bacterial strains. Due to the probable toxicity of produced metabolite following physicochemical or enzymatic treatment of synthetic dyes, many protocols have been developed for determination the toxicity of dyes and related compounds based on the growth inhibition of bacterial (*B. megaterium* and *E. coli*), yeast (*Saccaromyces cerevisiae*), or animal cells (human cervix cells; HeLa), and phytotoxicity studies on plant seeds (*Oryza sativa* or *Triticum aestivum*) [1,3]. In the present work, the toxicity of laccase-treated dye sample was significantly reduced compared to that of parent dye. Same results was reported by Palmieri et al. [28] who determined 95% viability for *B. cereus* in the presence of sample obtained from enzymatic elimination of Remazol Brilliant Blue R (an anthraquinone dye) using the purified laccase of *Pleurotus ostreatus*. The growth of *B. megaterium* and *E. coli* were reported to be 99% and 94%, respectively, in the presence of laccase-treated Malachite Green (a triphenylmethane dye). In general, azo dyes have been shown to be more resistant to enzymatic removal compared to other classes of synthetic dyes (like anthraquinone dyes) and provide higher toxicity after enzymatic removal [3,29]. However, Champagne and Ramsay [30] reported lower toxicity for azo dyes (Acid red 27 and Reactive black 5) compared to anthraquinone dyes (Reactive blue 19 and Dispersed blue 3) after treatment using free and immobilized laccases.

## Conclusion

Response surface methodology is an important tool to optimize the conditions for textile dye wastewater treatment. Such statistical approach reduces the number of runs and provides valuable information on possible interactions between the variables and response. The present study was designed to optimize the reaction condition for laccase- catalyzed decolorization of Acid Blue 92. The results indicated pH, enzyme activity, and dye concentration as the most important variables in the enzymatic decolorization of Acid Blue 92. Further studies should be performed on the decolorization of dyes from different families such as triphenylmethan, indigo, and anthraquinone dyes, extensively employing in the textile and chemical industries.
